# The Burden of Binge and Heavy Drinking on the Brain: Effects on Adolescent and Young Adult Neural Structure and Function

**DOI:** 10.3389/fpsyg.2017.01111

**Published:** 2017-06-30

**Authors:** Anita Cservenka, Ty Brumback

**Affiliations:** ^1^School of Psychological Science, Oregon State UniversityCorvallis, OR, United States; ^2^Mental Health Service, VA San Diego Healthcare SystemSan Diego, CA, United States; ^3^Department of Psychiatry, University of California, San DiegoSan Diego, CA, United States

**Keywords:** binge drinking, heavy drinking, adolescence, young adulthood, MRI and fMRI

## Abstract

**Introduction:** Adolescence and young adulthood are periods of continued biological and psychosocial maturation. Thus, there may be deleterious effects of consuming large quantities of alcohol on neural development and associated cognition during this time. The purpose of this mini review is to highlight neuroimaging research that has specifically examined the effects of binge and heavy drinking on adolescent and young adult brain structure and function.

**Methods:** We review cross-sectional and longitudinal studies of young binge and heavy drinkers that have examined brain structure (e.g., gray and white matter volume, cortical thickness, white matter microstructure) and investigated brain response using functional magnetic resonance imaging (fMRI).

**Results:** Binge and heavy-drinking adolescents and young adults have systematically thinner and lower volume in prefrontal cortex and cerebellar regions, and attenuated white matter development. They also show elevated brain activity in fronto-parietal regions during working memory, verbal learning, and inhibitory control tasks. In response to alcohol cues, relative to controls or light-drinking individuals, binge and heavy drinkers show increased neural response mainly in mesocorticolimbic regions, including the striatum, anterior cingulate cortex (ACC), hippocampus, and amygdala. Mixed findings are present in risky decision-making tasks, which could be due to large variation in task design and analysis.

**Conclusions:** These findings suggest altered neural structure and activity in binge and heavy-drinking youth may be related to the neurotoxic effects of consuming alcohol in large quantities during a highly plastic neurodevelopmental period, which could result in neural reorganization, and increased risk for developing an alcohol use disorder (AUD).

## Introduction

Magnetic resonance imaging (MRI) studies have highlighted ongoing brain maturation through young adulthood (Gogtay et al., 2004). Decreases in cortical gray matter (GM) from ages 10–12 through adulthood have been attributed to synaptic pruning, a process that prioritizes efficiency and strengthening of connections via proliferation of myelin over the creation of new synaptic connections that occurs in childhood (Amlien et al., 2016). White matter (WM) volume increases linearly through young adulthood, which yields relatively stable total brain volumes after puberty (Giedd et al., 2009). This period of significant cortical modification coincides with increases in behavioral risk taking including the use of alcohol and other substances.

Alcohol use has negative effects on cognition and the brain (Jacobus and Tapert, 2013) and on health and safety (Nhtsa, 2014), yet drinking in high quantities increases during adolescence as nearly 25% of high school seniors report getting drunk in the last 30 days (Johnston et al., 2017). Binge or heavy episodic drinking (i.e., 4 or more standard drinks within a 2 h drinking session for females, 5 or more drinks for males) (NIAAA, 2004)^1^ leads to increased risk for negative acute effects, such as drunk driving, unsafe sex, and other substance use (Miller et al., 2007). Long-term, adolescent alcohol use is related to serious psychosocial problems, including comorbid psychopathology (Deas and Thomas, 2002), poorer academic success (Kristjansson et al., 2013), and detrimental neurocognitive consequences (Jacobus and Tapert, 2013). Furthermore, binge drinking patterns initiated during late adolescence often persist into early adulthood (Degenhardt et al., 2013) and initiating heavy drinking at an early age significantly increases risk for subsequent adult alcohol use disorders (AUD) and related problems (Hingson et al., 2006).

Given the increase of binge and heavy drinking during adolescence when protracted brain maturation is still underway, understanding the potentially harmful effects of consuming large quantities of alcohol on neural development and associated cognition is of central importance. The purpose of this mini review is to highlight associations that may reflect deleterious effects of binge drinking and also to inform future investigations into the effects of binge drinking on brain development and functioning in young binge/heavy episodic drinkers (BD/HD). Thus, we excluded samples based on diagnostic criteria (e.g., alcohol abuse or AUD), treatment studies, and those that characterized drinking based on non-binge or heavy-drinking criteria (e.g., lifetime alcohol use days).

## Structural brain imaging

Structural MRI assesses the metrics (e.g., thickness, surface area, and volume) of specific brain tissues at the macrostructure level. Additional techniques utilize the diffusion of water molecules [e.g., diffusion tensor imaging (DTI)] to characterize the microstructure of GM and WM. The majority of studies present cross-sectional data using retrospective reports of drinking experience, while a few recent studies have reported longitudinal changes in brain structure associated with binge drinking (Table 1).

**Table 1:** Structural MRI findings in binge/heavy-drinking adolescents and young adults.

**Study**	**Population (N)**	**Age (Mean ± SD)**	**BD/HD criteria**	**LD/controls criteria**	**Main findings^a^**
**GRAY/WHITE MATTER MACROSTRUCTURE**					
Banca et al., 2016	BD: 30	BD: 21.9 ± 3.3	BDE at least once a week for the last 3 months	Drinking threshold not specified; Drinks per week = 4.8 ± 2.4	No between group differences in ROIs [cerebellum, DLPFC, inferior parietal, or thalamus] selected due to negative correlation with impulsivity
	C: 30	C: 22.2 ± 3.4			
Doallo et al., 2014	BD: 11	BD: 22.2 ± 1.1	Either (1) weekly six or more alcoholic drinks (10 g of alcohol)^e^, OR (2) monthly six or more alcoholic drinks (10 g of alcohol) with pace of 3 drinks per hour, for ≥3 years	Less than monthly drinking six or more alcoholic drinks (10 g of alcohol) and pace of no more than 2 drinks per hour, for ≥3 years	BD ↑ volume mid-DLPFC (BA46 and BA9) and ACC in ROI analysis; Speed and quantity of alcohol consumption ↑ correlation with mid-DLPFC volume; also ↑ volume in MOG (BA 19), ACC/MeFG (BA32), Precentral Gyrus/MFG (BA6), and MCG (BA24) in uncorrected whole brain analysis
	C: 21	C: 22.4 ± 1.0			
Heikkinen et al., 2017	HD:35	^b^HD: 21.9 ± 3.3	AUDIT-C score ≥4 (males) and ≥3 (females) over 10 years	AUDIT-C score ≤ 2 over 10 years	No group differences in whole-brain VBM analysis; HD ↓ volume in subgenual ACC, OFC, STG and IC in ROI analyses
	LD:27	^b^LD: 22.2 ± 3.4			
Howell et al., 2013	BD: 19	BD: 21.9 ± 3.3	BDE at least once a week for the last 3 months	Drinking threshold not specified; AUDIT Scores = 3.2 ± 2.7	BD ↑ volume VS, thalamus, and lingual gyrus; BD ↓ volume right precuneus
	C: 19	C: 22.2 ± 3.4			
Kvamme et al., 2016	BD: 30	BD: 21.1 ± 1.8	BDE at least once a week for the last 6 months	Drinking threshold not specified; AUDIT Scores = 4.0 ± 2.6	No main effect of group; Group × Sex interaction [male: BD < C/female: BD > C] fusiform gyrus, SMA, temporal middle lobe, frontal inferior operculum, postcentral gyrus, precuneus, caudate, and VS
	C: 46	C: 20.3 ± 1.3			
Lisdahl et al., 2013	BD: 46	BD: 18.0 ± 0.8	BDE at least once in the last 3 months	No BDE in the last 3 months	BD: Number of BDE was inversely related to cerebellar volume of gray and white matter
	C: 60	C: 17.7 ± 1.0			
Luciana et al., 2013	AI: 30	^b^AI: 19.2 ± 1.4	Initiated alcohol consumption over 2 year follow-up period	No alcohol consumption	AI ↓ cortical thickness in MFG; AI ↓ WM volume in precentral, lingual, cingulate gyri, and MFG
	C: 25	^b^C: 18.6 ± 1.3			
Mashhoon et al., 2014	BD: 23	BD: 22.0 ± 1.2	≥3 BDEs per month for last 3 months	1 to 2 drinks per week and no BDE in past 3.5 years	BD ↓ cortical thickness in ACC and PCC; BD: negative correlation between drinks per week and ACC cortical thickness
	LD: 31	LD: 21.5 ± 1.6			
Medina et al., 2007^c^	HD: 16	HD: 16.9 ± 0.7	Monthly drinking; Alcohol as only substance of use	<60 lifetime alcohol use days; no abuse/dependence diagnosis	HD ↓ left Hip volume and ↑ right/left Hip asymmetry
	C: 21	C: 17.5 ± 1.1			
Pfefferbaum et al., 2016	BD: 134	BD: 18.6 ± 2.0	At least 1 past year BDE	No lifetime BDE	BD ↓ volume in frontal and temporal cortices; BD ↓ thickness in frontal, temporal, and cingulate cortices; BD: past year binges negatively related to frontal and parietal cortical thickness
	LD: 674	LD: 15.2 ± 2.4			
Squeglia et al., 2012b	BD: 29	BD: 18.2 ± 0.8	≥1 BDE in past 3 months	<3 drinks total in the past 3 months; no lifetime BDE	No main effect of group; Group × Sex interaction [male: BD < C/female: BD > C] in frontal pole, left pars orbitalis, left medial OFG, and left rostral ACC
	LD: 30	LD: 18.0 ± 1.1			
Squeglia et al., 2014	HD: 20	^b^HD: 18.0 ± 2.0	See Figure 1 in (Squeglia et al., 2012a) for full criteria	Drink <1x/month with 1–2 drinks on average, <5 drinks maximum ever on one occasion	HD ↓ volume left VDC, ITG, MTG, caudate, and brain stem; HD: ↑ alcohol use days, ↓ caudate, brain stem volume
	C: 20	^b^C: 17.2 ± 1.6			
Squeglia et al., 2015	HD: 75	^b^HD: 19.6 ± 1.9	See Figure 1 in (Squeglia et al., 2015) for full criteria	Drink <1x/month with 1–2 drinks on average, <5 drinks maximum ever on one occasion	HD ↓ volume frontal, lateral frontal, and temporal cortices; HD = less increase in pons and CC WM
	C: 59	^b^C: 17.3 ± 2.0			
(Wilson et al., 2015)	96^d^	^b^16.4 ± 0.9	Drinking treated as continuous variable; 10% reported past year BDE at follow up		Drinking ↓ volume right MFG, right pars triangularis, left and right MTG, left and right ITG, Amyg and left VDC; Drinking ↑ volume cerebellum; Drinking ↓ thickness right SFG, right MFG, right pars triangularis, and left and right MTG;
**DIFFUSION TENSOR IMAGING MICROSTRUCTURE**					
Jacobus et al., 2009, ^c^	BD: 14	BD: 18.1 ± 0.7	≥1 BDE in past 3 months	<3 drinks total in the past 3 months	BD ↓ FA in right ILF, left IFOF, left middle cerebellar peduncle, left SLF, and 4 clusters in left SCR
	C: 14	C: 17.3 ± 0.8			
Jacobus et al., 2013^c^	BD: 17	^b^BD: 20.9 ± NR	≥3 BDE in past year	Minimal drinking experience and no history of BDE	BD ↓ FA in splenium, genu, IFOF, anterior thalamic radiations, uncinate fasciculus, SLF, anterior limb internal capsule, ACR, SCR, and posterior limb internal capsule; BD: FA decreased across 3 years (~18–21 years old)
	C: 16	^b^C: 20.9 ± NR			
Luciana et al., 2013	AI: 30	^b^AI: 19.2 ± 1.4	Initiated alcohol consumption over 2 year follow-up period	No alcohol consumption	AI ↓ FA in dorsal caudate and IFOF
	C: 25	^b^C: 18.6 ± 1.3			
Mcqueeny et al., 2009	BD: 14	BD: 18.1 ± 0.7	≥1 BDE in past 3 months	Minimal drinking experience and no history of BDE	BD ↓ FA in ACR, CC, SLF, posterior limb of internal capsule, external capsule, fornix / stria terminalis, inferior cerebellar peduncle, superior cerebellar peduncle, PCR, and ILF (18 clusters total)
	C: 14	C: 18.0 ± 0.9			
Morris et al., 2017	BD: 28	BD: 22.0 ± 4.5	BDE at least once a week for the last 6 months	No BDE in past 6 months	BD ↓ ODI in frontal cortical GM and ↑ ODI in parietal GM; BD ↑ neurite density in cortical WM in adjacent regions of lower ODI; BD: ↑ VS ODI positively correlated with binge history
	C: 38	C: 23.7 ± 3.9			

*ACC, anterior cingulate cortex; ACR, anterior corona radiata; AI, alcohol initiators; Amyg, amygdala; AUDIT, alcohol use disorders identification test; AUDIT-C, alcohol use disorder identification test – 3 item short form; BA, Brodmann's Area; BD, binge drinkers; BDE, binge drinking episode, defined as ≥5 drinks/occasion for males, ≥4 drinks/occasion for females; C, controls; CC, corpus callosum; DLPFC, dorsolateral prefrontal cortex; FA, fractional anisotropy; GM, gray matter; HD, heavy drinkers; Hip, hippocampus; IC, insular cortex; ILF, inferior longitudinal fasciculus; IFOF, inferior fronto-occipital fasciculus; ITG, inferior temporal gyrus; LD, light drinkers; MCG, middle cingulate gyrus; MOG, middle occipital gyrus; MFG, middle frontal gyrus; MeFG, medial frontal gyrus; MTG, middle temporal gyrus; ODI, orientation dispersion index; OFG, orbital frontal gyrus; PCC, posterior cingulate cortex; PCR, posterior corona radiata; ROI, region of interest; SCR, superior corona radiata; SFG, superior frontal gyrus; SLF, superior longitudinal fasciculus; SMA, supplementary motor area; STG, superior temporal gyrus; VDC, ventral diencephalon; VS, ventral striatum; WM, white matter; ↓, less or decreased; ↑, greater or increased*.

a *Relative to LD/controls, unless otherwise specified*.

b *Age at final assessment time point*.

c *A group with combined heavy alcohol and marijuana use is not included in the current summary*.

d *Study consists of 48 monozygotic twin pairs followed for 2 years*.

e *Six drinks with 10 g of alcohol is similar to NIAAA guidelines of 5 or more drinks with 0.6 ounces (~14 grams) of pure alcohol per drink*.

### GM and WM macrostructure

Several cross-sectional studies have examined brain structure and binge and heavy-drinking histories of varying lengths in young drinkers, and the majority have highlighted regions of interest where alcohol-related deficits have been identified in chronic alcoholics (Pfefferbaum et al., 1998). Many studies report smaller volumes or thinner tissue distributed across neocortical regions primarily in frontal cortices, but also in temporal and parietal cortices (see Table 1). For example, a study that followed drinking patterns of young adults for 10 years reported HD exhibited reduced GM volume in the anterior cingulate cortex (ACC), orbitofrontal cortex (OFC), temporal gyrus, and insular cortex compared to light drinkers (LD) (Heikkinen et al., 2017). One study targeting the ACC also reported decreased cortical thickness among BD compared to LD (Mashhoon et al., 2014), while another study found that BD exhibited larger ACC volumes (Doallo et al., 2014). A large cross-sectional study reported that BD (*n* = 134) exhibited smaller volumes and thinner cortical tissue in total, frontal, and temporal GM as well as thinner cingulate cortex compared to controls (*n* = 674). In addition, within the BD group the number of binges in the previous year was negatively related to frontal and parietal cortical thickness (Pfefferbaum et al., 2016).

Subcortical regions including the hippocampus, diencephalon, cerebellum and brain stem also exhibit decreased volume among BD. For example, smaller left hippocampal volume in conjunction with greater hippocampal asymmetry in BD compared to controls has been found (Medina et al., 2007). Other studies reported brain stem volumes were smaller in HD compared to LD (Squeglia et al., 2014), and binge drinking episodes were inversely related to cerebellar volume (Lisdahl et al., 2013). Conversely, one study reported increased volume in the ventral striatum and thalamus among BD compared to controls (Howell et al., 2013). Interestingly, two studies found no differences between BD compared to controls/LD, but discovered a BD by sex interaction such that male BD exhibited smaller volumes compared to male controls/LD in several frontal, temporal, and subcortical regions, while female BD had larger volumes than female controls/LD in the same regions (Squeglia et al., 2012b; Kvamme et al., 2016).

Two longitudinal studies were able to examine structural MRI changes in adolescents who had a pre-drinking baseline measure. One reported greater-than-expected decline in cortical thickness in the middle frontal gyrus (MFG) associated with the onset of binge drinking (Luciana et al., 2013), as well as greater increases in several distributed WM regions over 2 years in non-drinkers compared to BD (Luciana et al., 2013). In a larger sample similar accelerated declines in frontal and temporal cortical volumes in BD and slower increases in WM were reported (Squeglia et al., 2015). A co-twin study attempted to parse out effects of drinking from genetic (or other) pre-existing vulnerabilities by examining co-twin deviations, and reported that reduced volume of the ventral diencephalon and middle temporal gyrus could be attributed to drinking, while reduced volume of the right amygdala and increased volume of the left cerebellum appeared to be pre-existing vulnerability for the onset of drinking (Wilson et al., 2015).

Taken together, binge drinking appears to be largely associated with decreased volume and accelerated thinning in the frontal and prefrontal cortices and slowing of expected WM increases. Allocortical and subcortical regions may reflect some specific positive associations with binge drinking (e.g., ventral striatum), and there is some evidence that male and female BD may exhibit an inverse relationship in some frontal and subcortical regions.

### GM and WM microstructure

Among alcohol dependent adults WM integrity tends to be weakened (Pfefferbaum et al., 2006), but fewer studies have examined the effects of binge drinking on WM and GM microstructure (see Table 1). Each study among non-dependent BD has reported WM integrity deficits compared to LD/controls across the majority of WM tracts (Jacobus et al., 2009; Mcqueeny et al., 2009; Bava et al., 2013). Longitudinal studies also support decreased WM integrity among individuals who initiate or increase binge drinking, showing additional declines in fractional anisotropy over time (Jacobus et al., 2013; Luciana et al., 2013). A recent study examining both GM and WM microstructure utilizing orientation dispersion index (ODI) reported that BD had lower ODI in frontal GM but higher ODI in parietal GM and in the ventral striatum (Morris et al., 2017). Thus, overall it appears that binge drinking is associated with decreased WM microstructural integrity, but may be selectively related to increases in microstructural GM in a brain region associated with reward seeking.

## Functional magnetic resonance imaging (fMRI)

As structural abnormalities have been related to heavy alcohol use during neuromaturation, it is important to understand whether these findings translate to alterations in the functioning of brain systems across different cognitive domains. We discuss six areas that have included studies of BD/HD: response inhibition, working memory, verbal learning and memory, decision making and reward processing, alcohol cue reactivity, and socio-cognitive/socio-emotional processing (Table 2). Further, in order to focus this section of the mini review on task-related functional magnetic resonance imaging (fMRI) studies, we excluded discussion of functional connectivity (Gorka et al., 2013; Weiland et al., 2014; Morris et al., 2016), acute alcohol administration (Filbey et al., 2008), machine learning (Squeglia et al., 2017), treatment (Feldstein Ewing et al., 2016), and neurofeedback (Kirsch et al., 2016) studies that included young BD/HD, as well as studies where binge drinking was examined, but was not the main variable of interest (Glaser et al., 2014).

**Table 2:** fMRI findings in binge/heavy-drinking adolescents and young adults.

**Study**	**Population (N)**	**Age (Mean ± SD)**	**BD/HD criteria**	**LD/controls criteria**	**Main findings^a^**
**INHIBITORY CONTROL**					
Ahmadi et al., 2013	56 HD	HD: 19.0 ± 0.5	AUD or drank >half the weeks in preceding 6 months with BD pattern^b^	No current or past AUD; drank <half the weeks in preceding 6 months	HD: ↓ ACC, SMA, parietal, hippocampal, MFG, STG activity during correct NoGo
	36 LD	LD: 18.8 ± 1.0			
Ames et al., 2014	21 HD	HD: 20.2 ± 1.4	Males: ≥15 drinks/week	Expected to drink <3 times/week and consume ≤2 drinks/occasion	HD: ↑ DLPFC, ACC/MeFG, anterior insula activity during correct NoGo
	20 LD	LD: 20.8 ± 1.1	Females: ≥8 drinks/week		
			BD ≥twice/week^b^		
Campanella et al., 2016	19 HD	HD: 24.7 ± 3	≥8 on AUDIT	≤7 on AUDIT	HD: ↑ occipital lobe, amygdala activity during failed NoGo
	17 LD	LD: 25.8 ± 4.2			
Wetherill et al., 2013	20 HD	Baseline HD: 14.7 ± 1.1	See Figure 1 in Wetherill et al., 2013	See Figure 1 in Wetherill et al., 2013	HD: ↑ fronto-parietal and cerebellar activity at follow-up, but less at baseline for NoGo vs. Go
	20 LD	Baseline LD: 14.1 ± 1.2			
		Follow-up HD: 18.5 ± 1.8			
		Follow-up LD: 17.6 ± 1.2			
**WORKING MEMORY**					
Campanella et al., 2013	16 BD	BD: 20.9 ± 1.8	≥6 drinks/occasion at speed of ≥2 drinks/hour, <2–3 times/week	Drank 1–30 days/month, ≤5 drinks/occasion at speed ≤2 drinks/hour	BD: ↑ pre-SMA during working memory
	16 C	C: 21.6 ± 2.6			
Squeglia et al., 2011	40 BD	BD Males: 18.1 ± 0.7	BD pattern ≥once in 3 months before scan	<3 drinks in past 3 months	Female BD vs. Female C: ↓ frontal, cerebellar, temporal during SWM Male BD vs. Male C: ↑ frontal, cerebellar, temporal during SWM
	55 C	BD Females: 17.8 ± 1.0			
		C Males: 17.7 ± 1.0			
		C Females: 18.1 ± 0.9			
Squeglia et al., 2012a	20 HD	Baseline HD: 15.1 ± 1.3	See Figure 1 in Squeglia et al., 2012a	See Figure 1 in Squeglia et al., 2012a	HD: ↑ fronto-parietal activity from baseline to follow-up during VWM
	20 C	Baseline LD: 14.7 ± 1.1			
		Follow-up HD: 18.5 ± 1.9			
		Follow-up LD: 17.7 ± 1.4			
**VERBAL LEARNING/MEMORY**					
Schweinsburg et al., 2010	12 BD	BD: 18.1 ± 0.7	Typical BD pattern^b^	No drinking past three months (See details in Table 1 in Schweinsburg et al., 2010)	BD: ↑ SFG/MFG/SPL/IPL, ↓ occipital activity during novel encoding
	12 C	C: 17.6 ± 0.8			
Schweinsburg et al., 2011	16 BD	BD: 18.2 ± 0.8	Typical BD pattern^b^ in last three months	≤5 lifetime marijuana uses; ≤50 lifetime alcohol uses	BD: ↑ fronto-parietal, ↓ precuneus, cingulate, inferior frontal gyrus activity during novel encoding
	22 C	C: 17.8 ± 0.9			
Dager et al., 2014b	23 HD	HD: 18.9 ± 0.6	AUD or drank >half the weeks in preceding 6 months with BD pattern^b^	No current or past AUD; drank <half the weeks in preceding 6 months	BD: ↑ DLPFC, posterior parietal, IFG, hippocampal activity during correct encoding BD: ↓ insula during correct recognition
	33 LD	LD: 18.7 ± 0.4			
**DECISION MAKING/REWARD PROCESSING**					
Xiao et al., 2013	14 BD	BD: 17.3 ± 0.5	≥5 drinks/occasion at least once in past month	Alcohol-naïve	BD: ↑ amygdala and insula activity during decision making in IGT
	14 C	C: 17.1 ± 0.7			
Jones et al., 2016	13 BD	Baseline BD: 14.9 ± 1.2	≥one BD pattern and ≥3 total occasions of ≥4 drinks within the last 90 days	Alcohol and substance-naïve	BD: ↓ dorsal striatal activity at follow-up, ↓ fronto-parietal and temporal activity at baseline and revisit during risky vs. safe decision making
	13 C	Baseline C: 14.9 ± 1.1			
		Follow-up BD: 17.7 ± 1.2			
		Follow-up C: 17.0 ± 1.1			
Worbe et al., 2014	19 BD	BD: 23.2 ± 3.5	>8 drinks for males (>6 for females) in a 2-h period, at least once a week, over a period of 3 months	Not described	BD: ↑ IFG activity associated with decrease in risky choices when feedback presented during risky-choice task
	21 C	C: 24.1 ± 3.1			
Cservenka et al., 2015	17 BD	Baseline BD: 14.9 ± 1.1	≥one BD pattern and ≥2 total occasions of ≥4 drinks within the same 90 day period	Alcohol and substance-naïve	BD: ↓ cerebellar activity during win vs. no win reward processing
	17 C	Baseline C: 14.8 ± 0.8			
		Follow-up BD: 16.9 ± 1.3			
		Follow-up C: 16.7 ± 1.2			
**ALCOHOL CUE REACTIVITY**					
Dager et al., 2013	35 HD	FHP HD: 19.3 ± 0.8	AUD or drank >half the weeks in preceding 6 months with BD pattern^b^	No current or past AUD; drank <half the weeks in preceding 6 months	BD: ↑ limbic, visual, frontal, and insular activity to alcohol vs. non-alcohol cues
	30 LD	FHN HD: 19.2 ± 0.7			
		FHP LD: 18.9 ± 1.0			
		FHN LD: 19.4 ± 0.6			
Kreusch et al., 2015	12 HD	21.3 ± 2.1	15 drinks/week on average ≥1 binge (i.e., 6 or more standard Dutch units of alcohol of 10 g each on 1 occasion) per week	N/A	HD: ↑ VTA activity in presence of task-irrelevant alcohol vs. soft-drink cues
Dager et al., 2014a	16 HD 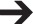 HD	HD 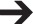 HD: 18.7 ± 0.8	HD: BD pattern^b^ ≥13 of the past 26 weeks, and averaging ≥30 drinks/month (7 drinks/week) in the 6 months before scanning. MD: BD pattern <13 of the previous 26 weeks, averaging ≤30 drinks/month, and never meeting criteria for AD.	N/A	MD 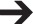 HD: ↑ fronto-striatal and insular activity to alcohol vs. non-alcohol cues
	14 MD 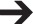 HD	MD 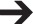 HD: 18.2 ± 0.4			
	13 MD 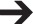 MD^c^	MD 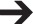 MD: 18.5 ± 0.9			
Brumback et al., 2015	32 HD	HD: 17.9 ± 0.7	≥100 lifetime drinking episodes, ≥3 past month BD pattern^b^ (≥1 in 2 weeks before study), and ≥1 recent alcohol withdrawal symptoms	<5 lifetime drinking episodes, no history of BD pattern or alcohol withdrawal symptoms	HD: ↓ differences in ACC and cerebellar activity to alcohol vs. non-alcohol cues after 1 month of abstinence
	19 C	C: 17.4 ± 0.7			
**SOCIO-COGNITIVE/SOCIO-EMOTIONAL PROCESSING**					
Maurage et al., 2013	12 BD	BD: 24.2 ± 4.5	>5 doses of 10 g of pure ethanol on drinking day; >3 occasions/week; >2 doses/hour	<2 doses of 10 g of pure ethanol on drinking day; <1 occasion/week; <1 dose/hour	BD: ↓ in bilateral STG, ↑ in right MFG
	12 C	C: 23.4 ± 4.2			

ACC, anterior cingulate cortex; AD, alcohol dependence; Amyg, amygdala; AUD, alcohol use disorder; AUDIT, alcohol use disorders identification test; BD, binge drinkers; BOLD, blood oxygen level-dependent; C, controls; DLPFC, dorsolateral prefrontal cortex; FHN, family history negative; FHP, family history positive; HD, heavy drinkers; IFG, inferior frontal gyrus; IGT, Iowa Gambling Task; IPL, inferior parietal lobule; LD, light drinkers; MD, moderate drinkers; MFG, middle frontal gyrus; MeFG, medial frontal gyrus; MTG, middle temporal gyrus; SFG, superior frontal gyrus; SMA, supplementary motor area; SPL, superior parietal lobule; STG, superior temporal gyrus; SWM, spatial working memory; VTA, ventral tegmental area; VWM, verbal working memory; ↓, less or decreased; ↑, greater or increased

a *Relative to the LD/controls, unless otherwise specified*.

b *BD pattern, ≥5 drinks/occasion for males, ≥4 drinks/occasion for females*.

c *Three groups included participants who started as heavy drinkers at baseline and remained heavy drinkers at follow-up (HD 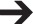 HD), participants who were moderate drinkers at baseline and transitioned into heavy drinking at follow-up (MD 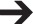 HD), and participants who started as moderate drinkers at baseline and remained moderate drinkers at follow-up (MD 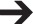 MD)*.

### Response inhibition

The ability to inhibit a pre-potent response or have self-control over impulsive actions is a central facet of executive functioning (Diamond, 2013). Several studies have identified deficits in response inhibition and its neural correlates in individuals with AUD (Lawrence et al., 2009), and these investigations have extended to adolescent and young adult BD/HD, most of which have used Go/NoGo tasks. For example, in a study of 18–20 year old college students, HD showed slower reaction times on both correct Go hits and incorrect NoGo false alarms (Ahmadi et al., 2013). LD had greater response in ACC, supplementary motor area (SMA), MFG, parietal lobe, hippocampus, and superior temporal gyrus (STG) than HD during NoGo correct rejections, suggesting decreased inhibitory control brain activity in HD in a set of brain regions that underlie cognitive and impulse control (Ahmadi et al., 2013).

Variations of the Go/NoGo task have used alcohol-related images as NoGo stimuli and non-alcoholic beverages as Go stimuli. Ames et al. (2014) demonstrated that compared with HD, LD had better Go/NoGo task performance as indexed by *d*-prime. HD had greater activity in the dorsolateral prefrontal cortex (DLPFC), ACC, and the anterior insula than LD during NoGo trials, suggesting greater reliance on executive functioning, error monitoring, and emotional interoception regions during inhibitory control (Ames et al., 2014). Another task presented the traditional letters used in Go/NoGo tasks overlaid onto black, neutral picture, and alcoholic photo backgrounds. While there were no effects of background context, college HD displayed greater activity in visual and emotional processing regions, such as the amygdala and occipital lobe during *failed inhibitions* compared with LD (Campanella et al., 2016).

In one longitudinal investigation, HD had greater fronto-parietal and cerebellar activity during response inhibition relative to controls at follow-up but reduced activity in these same regions at baseline, suggesting both markers of vulnerability toward heavy drinking and altered executive functioning activity after the initiation of heavy alcohol use (Wetherill et al., 2013). Task-related fMRI studies have largely reported that HD/BD have increased fronto-parietal and cerebellar response during successful inhibitory control and increased emotional and visual response during unsuccessful response inhibition (except for Ahmadi et al., 2013).

### Working memory

Another key component of executive functioning is working memory (WrkM), the ability to maintain and manipulate information during a short time span (Diamond, 2013). WrkM has been linked with adaptive decision making and deficits in WrkM are associated with vulnerability toward addiction (Nagel et al., 2012). An fMRI n-back task of WrkM was completed by university BD, who showed larger pre-SMA WrkM-related activity than controls, suggesting greater attentional resources devoted to performing the task by the BD to maintain equal performance with the control group (Campanella et al., 2013).

Some studies have reported that sex differences may also be present in WrkM-related activation between male and female BD. Female BD had less spatial WrkM activation in several frontal, temporal, and cerebellar regions compared to female controls and this was linked to poor behavioral performance in the BD, a pattern opposite to what was seen in male BD relative to male controls (Squeglia et al., 2011). The authors argue that this may suggest female vulnerability toward the neurotoxic effects of binge drinking during active periods of neuromaturation.

While longitudinal research is sparse among fMRI studies of BD/HD youth, one study reported reduced baseline fronto-parietal activity in adolescents who later transitioned into heavy drinking. However, HD showed significantly increased activity in these areas at a 3-year follow-up relative to baseline brain response (Squeglia et al., 2012a). Overall, these studies suggest mostly greater WrkM-related brain activity across fronto-parietal regions in BD/HD relative to controls, but some exceptions may be present when examining sex differences and pre-drinking vulnerability.

### Learning and memory

Deficits in learning and memory have been previously reported in individuals with AUD (Pitel et al., 2014), and in investigations of BD youth (Carbia et al., 2017). In the first of three studies examining neural response during verbal or figural encoding, Schweinsburg et al. (2010) found that while learning novel word pairs, BD showed elevated superior frontal and posterior parietal activity compared with controls, a finding that was closely replicated in a subsequent study where BD had greater fronto-parietal activity during novel encoding, with some areas displaying reduced activity relative to controls, such as the inferior frontal gyrus (IFG), precuneus, and ACC (Schweinsburg et al., 2011). These findings suggest some degree of neural reorganization in BD that results in increased reliance on fronto-parietal regions while learning novel word pairs, and decreased activity in other regions.

Pictorial as opposed to verbal stimuli were used in a study of college HD who demonstrated similar patterns of brain activity to previous studies of adolescents, namely greater fronto-parietal activity during encoding of novel stimuli, as well as greater hippocampal response relative to LD (Dager et al., 2014b). This study also examined brain activity associated with recognition for the first time, and found less insular activity during correct recognition in HD vs. LD, a finding the authors believed could reflect less arousal during correct recognition or a different task approach that resulted in similar task performance (Dager et al., 2014b).

### Decision making and reward processing

A number of studies have investigated the neural correlates of risky decision making and reward processing across monetary decision making tasks in young BD. A study using the Iowa Gambling Task found that compared with their peers, adolescent BD had greater insular and amygdala activity, suggesting greater emotion-driven decision making in the BD (Xiao et al., 2013), but this task did not permit the dissociation of decision making-related activation from reward processing. A subsequent longitudinal study used a modified Wheel of Fortune Task, in which BD showed reduced dorsal striatum activity during risky vs. safe decision making, and similar to previous studies, reductions in fronto-parietal activity preceded the onset of heavy drinking (Jones et al., 2016). It is possible that feedback during risk taking could modify behavior and cognitive control as young adult BD decreased their risk taking when they were presented with information about potential monetary losses, and this was associated with increased recruitment of IFG (Worbe et al., 2014). Finally, processing of reward receipt was related to decreased cerebellar activity in a longitudinal study of BD, suggesting blunted reward and affect-related responses as a result of heavy episodic drinking (Cservenka et al., 2015). Based on these results, a general pattern that is emerging is related to alterations in cognitive control and emotional processing brain regions that may be modifiable when feedback about the consequences of risk taking are presented.

### Alcohol cue reactivity

Alcohol cue reactivity studies have found greater neural response in reward and emotional processing brain regions among individuals with AUD (Heinz et al., 2009). Alterations in motivational neurocircuitry are associated with AUD (Koob and Volkow, 2010) and have thus been investigated in young adult and adolescent BD/HD. Dager et al. (2013) reported that young adult HD had greater neural activity in response to alcohol-related images in widespread areas comprised of limbic, visual, frontal, and insular regions compared with LD. Further, in a task where participants were instructed not to focus on alcohol cues, ventral tegmental area activation was elevated in young adult HD compared with neural response seen to soft drink cues, suggesting automatic processing of alcohol-related stimuli that may increase motivational drive in mesolimbic circuitry (Kreusch et al., 2015). Interestingly, response to alcohol cues may be used to predict drinking behavior in young adult HD as those who showed elevated response in fronto-striatal areas and the insula subsequently transitioned into heavy drinking (Dager et al., 2014a). A longitudinal study of adolescent HD showed that increased brain activity to alcohol cues in HD vs. controls diminishes with abstinence from alcohol, indicating that a decline in risky drinking may modify brain activity in response to alcohol-related stimuli (Brumback et al., 2015). Across these studies, there is evidence that mesolimbic and motivational circuitry may be important targets for studies designed to reduce response to alcohol cues in adolescent and young adult HD.

### Socio-cognitive and socio-emotional processing

Research on the effects of binge and heavy drinking on the developing brain are limited in other domains, such as socio-cognitive and socio-emotional processing. While recent meta-analyses highlight deficits in social cognition in individuals with AUD (Onuoha et al., 2016; Bora and Zorlu, 2017), there are a lack of fMRI studies in this area within young BD/HD. In one study, young adult BD categorizing vocal affective stimuli had less activity in STG, but more activity in MFG compared with their peers (Maurage et al., 2013). Given the large gap in the literature specifically focused on socio-cognitive processing in young BD/HD, future research should further investigate this domain.

## Conclusions

Binge drinking among youth is associated with smaller/thinner cortical and subcortical structures and decreased WM integrity. Consistent across many fMRI studies of cognitive control, WrkM, and verbal learning, young BD and HD show greater reliance on fronto-parietal systems while performing these tasks (Schweinsburg et al., 2010, 2011; Squeglia et al., 2012a; Wetherill et al., 2013; Dager et al., 2014b). Executive functioning and emotional processing systems are important networks for future investigations related to decision making and reward processing (Xiao et al., 2013; Worbe et al., 2014; Cservenka et al., 2015; Jones et al., 2016), while mesolimbic circuitry is likely involved in the elevated response to alcohol cues in young BD/HD (Dager et al., 2013, 2014a; Brumback et al., 2015; Kreusch et al., 2015). These findings suggest there may be neural alterations as a result of heavy alcohol use or neural risk markers related to vulnerability toward heavy drinking during adolescence and young adulthood. While some findings have been replicated, greater efforts are needed for consistency across task variations, analyses reported, inclusionary criteria for BD/HD, as well as longitudinal studies of this topic.

## Author contributions

AC conducted literature searches, wrote, edited, and revised the section on fMRI findings, wrote the conclusions, and created the table of fMRI findings. TB conducted literature searches, wrote, edited, and revised the section on structural MRI findings, wrote the introduction, and created the table of MRI structural findings. AC edited the final version of the manuscript and wrote the abstract.

### Conflict of interest statement

The authors declare that the research was conducted in the absence of any commercial or financial relationships that could be construed as a potential conflict of interest.

## Footnotes

^1^While the definition of a standard drink differs by location outside of the United States (Mongan and Long, 2015). binge drinking episodes result in blood alcohol concentrations (BAC) near.08 gram percent (i.e., minimum of 2–3 ounces or 60–85 grams of pure alcohol).
